# MarvelD3 regulates the c-Jun N-terminal kinase pathway during eye development in *Xenopus*

**DOI:** 10.1242/bio.018945

**Published:** 2016-11-15

**Authors:** Barbara Vacca, Elena Sanchez-Heras, Emily Steed, Maria S. Balda, Shin-Ichi Ohnuma, Noriaki Sasai, Roberto Mayor, Karl Matter

**Affiliations:** 1 Institute of Ophthalmology, University College London, London EC1V 9EL, UK; 2 Division of Biomedical Sciences, Developmental Biomedical Science Laboratory, Graduate School of Biological Sciences, Nara Institute of Science and Technology (NAIST), 8916-5, Takayama-cho, Ikoma 630-0192, Japan; 3 Department of Cell and Developmental Biology, University College London, Gower Street, London WC1E 6BT, UK

**Keywords:** MarvelD3, *Xenopus* eye, Tight junction, C-Jun N-terminal Kinase

## Abstract

Ocular morphogenesis requires several signalling pathways controlling the expression of transcription factors and cell-cycle regulators. However, despite a well-known mechanism, the dialogue between those signals and factors remains to be unveiled. Here, we identify a requirement for MarvelD3, a tight junction transmembrane protein, in eye morphogenesis in *Xenopus*. MarvelD3 depletion led to an abnormally pigmented eye or even an eye-less phenotype, which was rescued by ectopic MarvelD3 expression. Altering MarvelD3 expression led to deregulated expression of cell-cycle regulators and transcription factors required for eye development. The eye phenotype was rescued by increased c-Jun terminal Kinase activation. Thus, MarvelD3 links tight junctions and modulation of the JNK pathway to eye morphogenesis.

## Abstract (precis)

**Summary:** MarvelD3 regulates eye morphogenesis through modulation of the JNK pathway, eye-field specification and the balance between cell proliferation and survival.

## INTRODUCTION

Eye morphogenesis is an evolutionarily conserved feature of most organisms to ensure an efficient communication with the environment (Gilbert, 2000). Eye development is a complex multi-step process, which involves cell proliferation, migration, cell-fate determination, survival and differentiation (Fuhrmann, 2008; Gilbert, 2000; Harada et al., 2007). After the neural antero-posterior patterning of the embryo, several signals, such as bone morphogenetic protein (BMP), fibroblast growth factor (FGF) and non-canonical Wnt, promote the expression of eye-field transcription factors (EFTFs). EFTFs specify a single eye-field in the most anterior region of the neural plate. In this region, inhibition of cell-cycle activators occurs to favour EFTF expression, while duration of the expression of the transcription factors is established by cell-cycle-independent factors (Bilitou and Ohnuma, 2010; Cavodeassi et al., 2005; Gilbert, 2000; Harada et al., 2007; Zuber, 2010). The single eye-field is then divided into two eye primordia under the influence of Sonic hedgehog signalling (Chiang et al., 1996; Gilbert, 2000; Roessler et al., 1996; Takabatake et al., 2002). These eye primordia are characterized by a highly mitotic activity due to an enhanced expression of cell-cycle activators. This proliferative step, driven by cyclins and cyclin-dependent kinases, allows the appropriate number of retinal precursors to be reached (Bilitou and Ohnuma, 2010; Harada et al., 2007). During this process, the optic vesicle invaginates and reaches the surface ectoderm. The dialogue between the optic vesicle and the ectoderm then specifies the lens placode, which is internalized and induces the development of the cornea from the surface ectoderm (Bilitou and Ohnuma, 2010; Gilbert, 2000; Harada et al., 2007; Zuber, 2010). In the optic vesicle, the fate of the retinal precursors is determined during the last-division step before their final differentiation (Bilitou and Ohnuma, 2010; Zuber, 2010). A non-canonical Wnt signal is then subsequently required for the final differentiation of the retinal progenitors into the cell types that make up the inner and outer retinal cell layers: glia, ganglion cells, interneurons, photoreceptors and the retinal pigment epithelium (RPE) (Gilbert, 2000; Harada et al., 2007; Maurus et al., 2005).

Most of the main eye components are derived from epithelia. Epithelial integrity is preserved by tight junctions (TJ), adherens junctions (AJ) and desmosomes (Balda and Matter, 2008; Eckert and Fleming, 2008; Fleming et al., 2000a,b; Ripley et al., 2004; Wei and Huang, 2013). MarvelD3 (MD3), a TJ transmembrane protein, belongs to the tight junction-associated MARVEL domain protein family, which also includes occludin and tricellulin (Cording et al., 2013; Raleigh et al., 2010; Shen et al., 2011; Steed et al., 2009). In the eye, occludin and tricellulin are thought to contribute to the compartmentalisation of the ocular micro-environments by controlling the flux through the retinal-blood barrier (Erickson et al., 2007; Huet et al., 2011; Iwamoto et al., 2014; Muthusamy et al., 2014; Tserentsoodol et al., 1998). Our previous work showed MD3 expression in corneal and RPE-derived cell lines (Steed et al., 2009). Additionally, MD3-mediated c-Jun N-terminal kinase (JNK) inhibition modulates cell proliferation, migration and survival *in vitro* (Steed et al., 2009). However, the function of MD3 in eye development has not been explored. Given the role of MD3 in the regulation of the JNK pathway, analysing its function in eye morphogenesis may lead to new insights into eye morphogenesis and the role of TJ-associated signalling mechanisms. We speculated that MD3 plays a role in the establishment of the complex regulatory systems that mediate eye morphogenesis and in the spatio-temporal regulation of the interplay between expression of EFTFs and cell-cycle regulators (Bilitou and Ohnuma, 2010; Zuber, 2010).

Here, we asked whether MD3 is involved in eye morphogenesis in *Xenopus*. We show that MD3 is expressed in the eye region similarly to JNK and that MD3 depletion disrupted the normal expression pattern of EFTFs, the balance between cell proliferation and survival, and led to the development of smaller eyes or to an eye-less phenotype. Similarly, MD3 overexpression affects the expression patterns of EFTFs, cell proliferation and survival but, contrary to MD3 depletion, overexpression led to no visible eye phenotype except a more compact lamination of the retina. These findings support a new role for MD3 as a regulator of ocular morphogenesis. Unexpectedly, the eye phenotype was not caused by increased JNK signalling but could be rescued by stimulating the JNK pathway in MD3-depleted embryos. Our data suggest that MD3 acts as a linker between the signals inducing eye morphogenesis, specification of the eye field by EFTFs, and survival and differentiation of multiple ocular cell types.

## RESULTS

### Structure and expression of MarvelD3 in the eye field

**Fig. 1.**
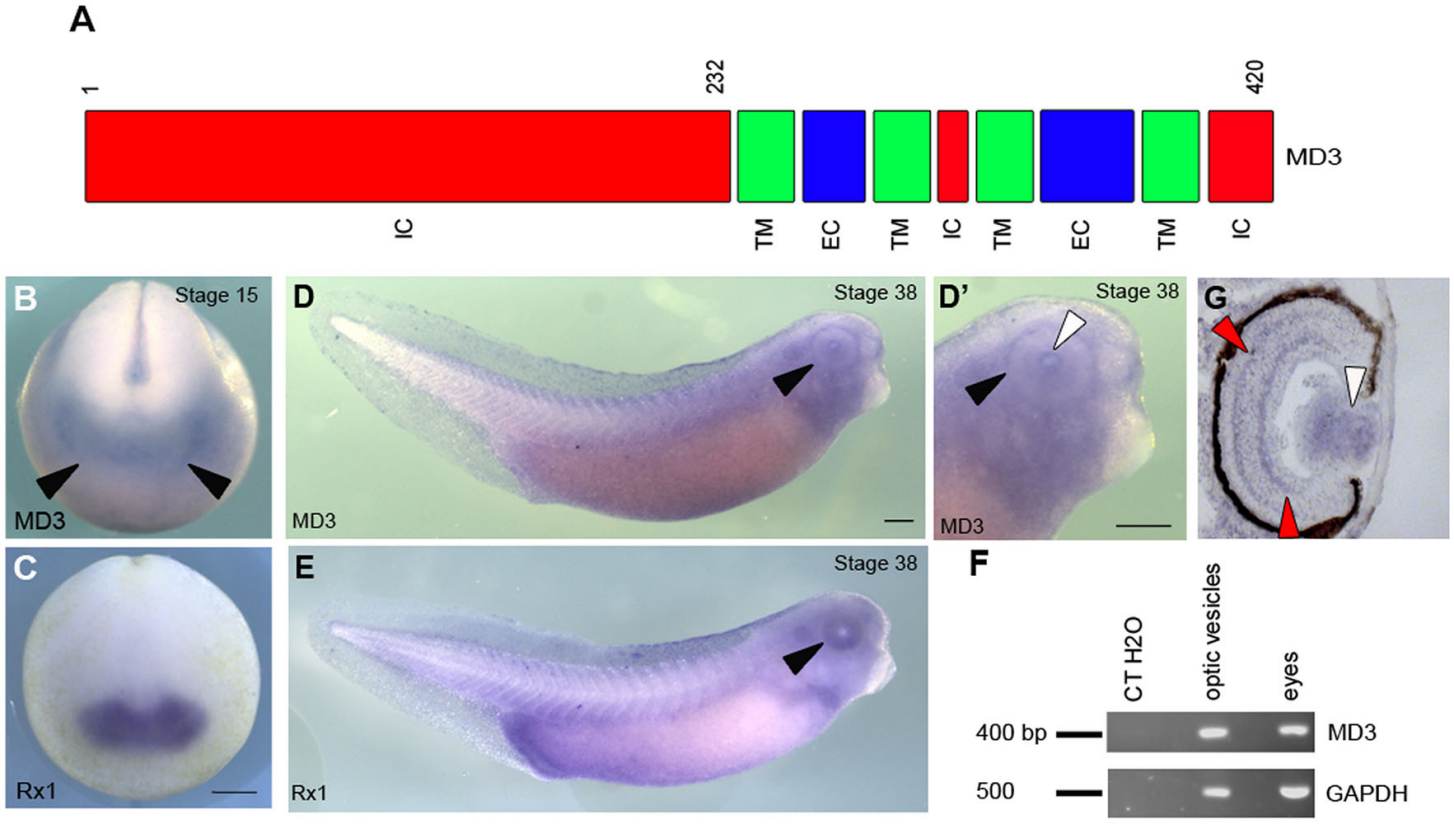
**MD3 structure and expression in *Xenopus* embryo.** (A) *Xenopus laevis* MD3 (MD3; Genbank: BC 068841) protein includes three intracellular (IC, red), four transmembrane (TM, green) and two extracellular (EC, blue) domains. Numbers correspond to the amino acids. Spatial analysis of expression of MD3 transcript (B,D,D′) and an eye marker Rx1 (C,E) was performed by whole-mount *in situ* hybridization (WISH) in neurulas (stage 15, B) and tadpoles (stage 38, D,D′). Scale bar: 500 μm; black arrowhead, eye; white arrowhead, lens. (F) MD3 expression analysis by semi-quantitative RT-PCR using RNA isolated from dissected optic vesicles (stage 25) and eyes (stage 42). GAPDH was amplified as a control. bp, base pairs. (G) MD3 signal was detected by WISH in the inner and outer plexiform layers (red arrowheads) and in the lens (white arrowhead) on eye sections (stage 42).

*Xenopus* MD3 (GenBank accession number, BC 068841) encodes a 420 amino acid (AA) protein with an amino-terminal intracellular domain followed by four transmembrane domains, two extracellular loops and an 18 AA cytoplasmic carboxy-terminal domain (Fig. 1A). *Xenopus* MD3 closely resembles isoform 2 of human MD3 (Steed et al., 2009). We first analysed the spatial distribution of MD3 expression by whole-mount *in situ* hybridization (WISH) and found that the MD3 transcript is broadly expressed in the animal pole before gastrulation (Fig. S1A,B). At the neurula stage, MD3 is enriched in the anterior region of the embryo, similarly to Rx1 (Retina homeobox 1), an eye marker (stage 15; Fig. 1B,C). Consistently, at tail-bud stages, MD3 is expressed in the head region (stage 25; Fig. S1C and Fig. 1D,D′), partially overlapping with Rx1 expression (Fig. 1E). To further substantiate our data on MD3 expression in the eye, we performed a RT-PCR analysis of the transcript using RNA purified from isolated optic vesicles (stage 25) and eyes (stage 42). Consistently with the results from the spatial analysis of MD3 expression, the transcript was detected in RNA derived from the eye field at both stages (Fig. 1F).

We previously showed that MD3 acts as an inhibitor of the JNK pathway (Steed et al., 2014), and MD3 distribution in *Xenopus* embryos is reminiscent of that of JNK and JNK activators (Fuhrmann, 2008; Hirai et al., 2005; Kibardin et al., 2006; Kim et al., 2005; Yamanaka et al., 2002). To determine in which region of the eye MD3 is expressed, we sectioned labelled embryos and detected MD3 expression in the lens, and in the inner and outer plexiform layers (Fisher, 1976) (Fig. 1G). MD3 is thus expressed in the head and, particularly, in specific tissues and cell layers of the developing eye.

### MD3 is required for the eye morphogenesis

To determine the functional importance of MarvelD3 for eye development, we designed two morpholino (MO) antisense oligonucleotides that block MD3 translation, one targeting the 5′-UTR (untranslated region; MD3A MO) and the other at the start of the ORF (open reading frame; MD3B MO) of MD3. Inhibition of MD3 expression by MD3 MO was confirmed by immunoblotting (Fig. S2A).

**Fig. 2.**
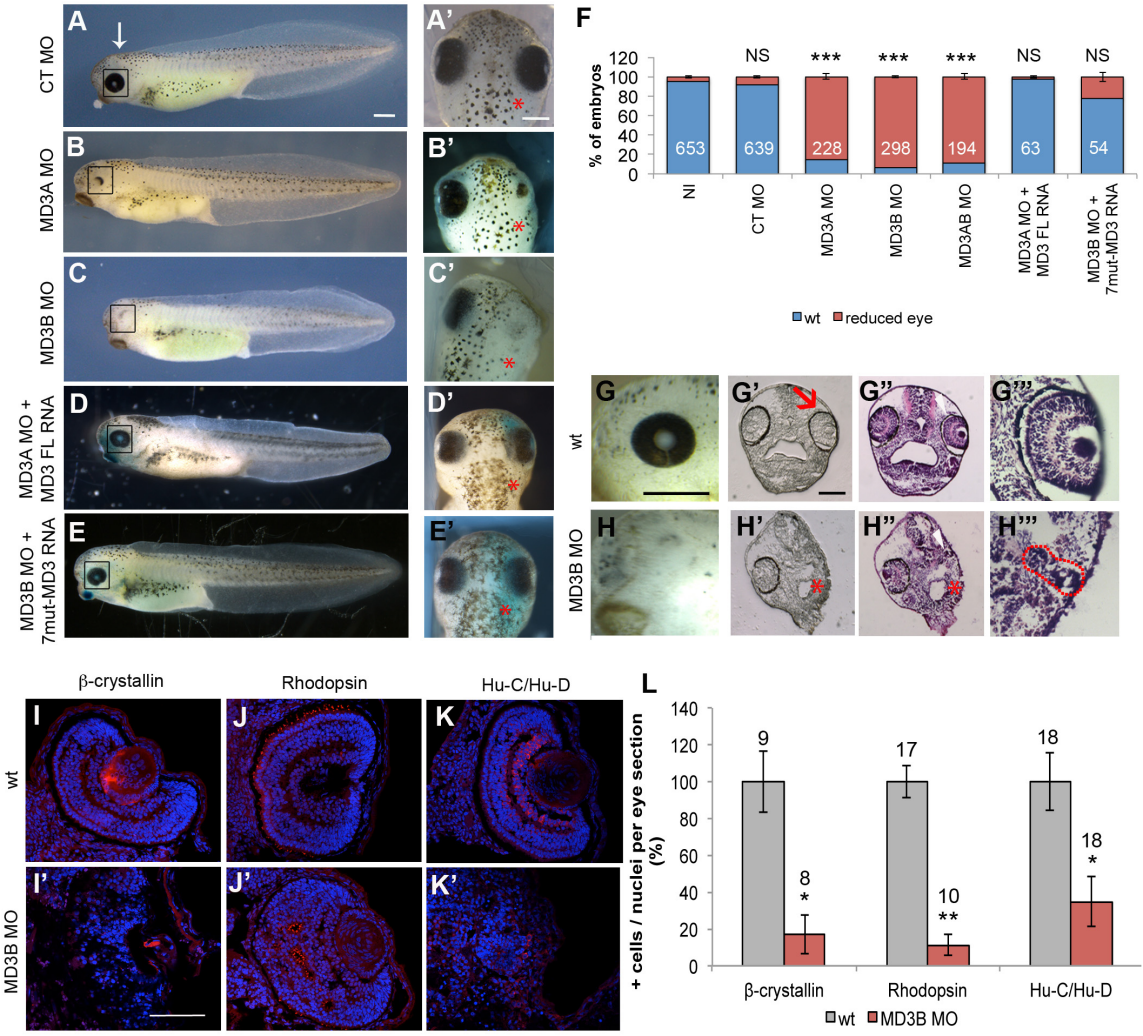
**MD3 KD disrupts eye morphogenesis.** Alteration of eye morphology was analysed (A-E′) and quantified (F) in non-injected (NI), CT, MD3A, MD3B, MD3AB MO-injected embryos and embryos co-injected with MD3A MO and FL MD3 mRNA or MD3B MO and 7-mut MD3 mRNA. Lateral (A-E) and dorsal view (A′-E′) of the embryo; white arrow, level for dorsal view; black rectangle, eye; red asterisk, injected side of the embryo in all figures. Numbers indicate the embryos analysed. Crop of the eye (G-H) and head transversal section without (G′-H″) or with a hematoxylin & eosin (H&E) staining (G′″-H′″) were analysed in wild-type (wt) and MD3B MO embryos. Red arrow, retinal pigment epithelium (RPE); white arrowhead, eye region; dotted red line, remaining eye tissue. Analysis by immunohistochemistry on eye sections from wt and MD3B MO-injected embryos was performed for lens cells (β-crystallin, I-I′), rod photoreceptors (Rhodopsin; J,J′) and ganglion cells (Hu-C/Hu-D, K-K′), and the number of positive cells for each eye marker was quantified as described in the Materials and methods (L); numbers indicate the eye sections quantified. Statistical significance was assessed by Student's *t*-test in F (*P* values, CT MO=0.14; MD3A MO=8.99*10^−5^; MD3B MO=2.21*10^−5^; MD3AB MO=2.74*10^−6^; MD3A MO+MD3 FL RNA=0.18; MD3B MO+7mut-MD3 RNA=6*10^−2^, NS: non significant; ***P<0.001), and Mann–Whitney test in L (*P* values, β-crystallin=1.59*10^−2^; Rhodopsin=1*10^−4^; Hu-C/Hu-D=1.92*10^−2^, *P<0.05 and **P<0.01). Scale bars: 500 µm (A-E,G,H) and 100 μm (I-K). Embryos were analysed at stage 42.

First, we asked whether MD3 MOs cause morphological changes during embryogenesis by injecting the dorsal animal region of a blastomere at the 2-cell stage. During gastrulation and neurula stages, no apparent morphological defects were observed (Fig. S2B-C). In contrast, at tailbud stage, injection of MO either individually or together (MD3A MO, 85.4±2.66%; MD3B MO, 93.9±1.04%; MD3AB MO, 88.7±2.96%) showed a patchy or absent retinal pigmentation, while control (CT) MO-injected embryos (91.5±1.26%) presented an intact ocular pigmentation (Fig. 2A-C and F), supporting the specificity of the MO-induced eye phenotype. To further substantiate the specificity of the observed phenotype, we performed rescue experiments by co-injecting MO with corresponding non-targeted mRNA. For MD3A MO, which targets a sequence in the 5′-UTR, we co-injected mRNA representing the coding region of MD3 (MD3 FL); and with MD3B MO, which binds at the start of the coding sequence, the mRNA encoded a mutated form carrying silent mutations in the MD3B MO targeting sequence (7mut-MD3). Both rescue approaches resulted in recovery of normal retinal pigmentation (97.4%±0.43 and 78.1%±1.63, respectively), suggesting the restoration of apparently normal eye development (Fig. 2D-E′ and F). Thus, the phenotype induced by MD3 MOs is specific and indicates that MD3 is required for eye development.

We then performed a more detailed investigation of the ocular structure to identify cell types affected in MD3 morphants. Histological analysis revealed a strong disorganisation of the overall eye structure, loss of retinal lamination, and absence of RPE (black pigmented ring) in MD3 MO injected embryos (Fig. 2G-H). Staining of sections for markers of specific ocular cell types further corroborated a retinal defect (Fig. 2I-K). Quantification of such images revealed reduced numbers of lens epithelial cells positive for β-crystallin (15.99%±6.7), ganglion cells positive for Hu-C/Hu-D (60.9%±15.38), and rod photoreceptors positive for rhodopsin (8.75%±4.6). Moreover, the remaining rod photoreceptors formed mislocalized clusters (Fig. 2I-L). MD3 is thus required for eye development and, in particular, for the formation of a laminated retina and differentiated lens.

**Fig. 3.**
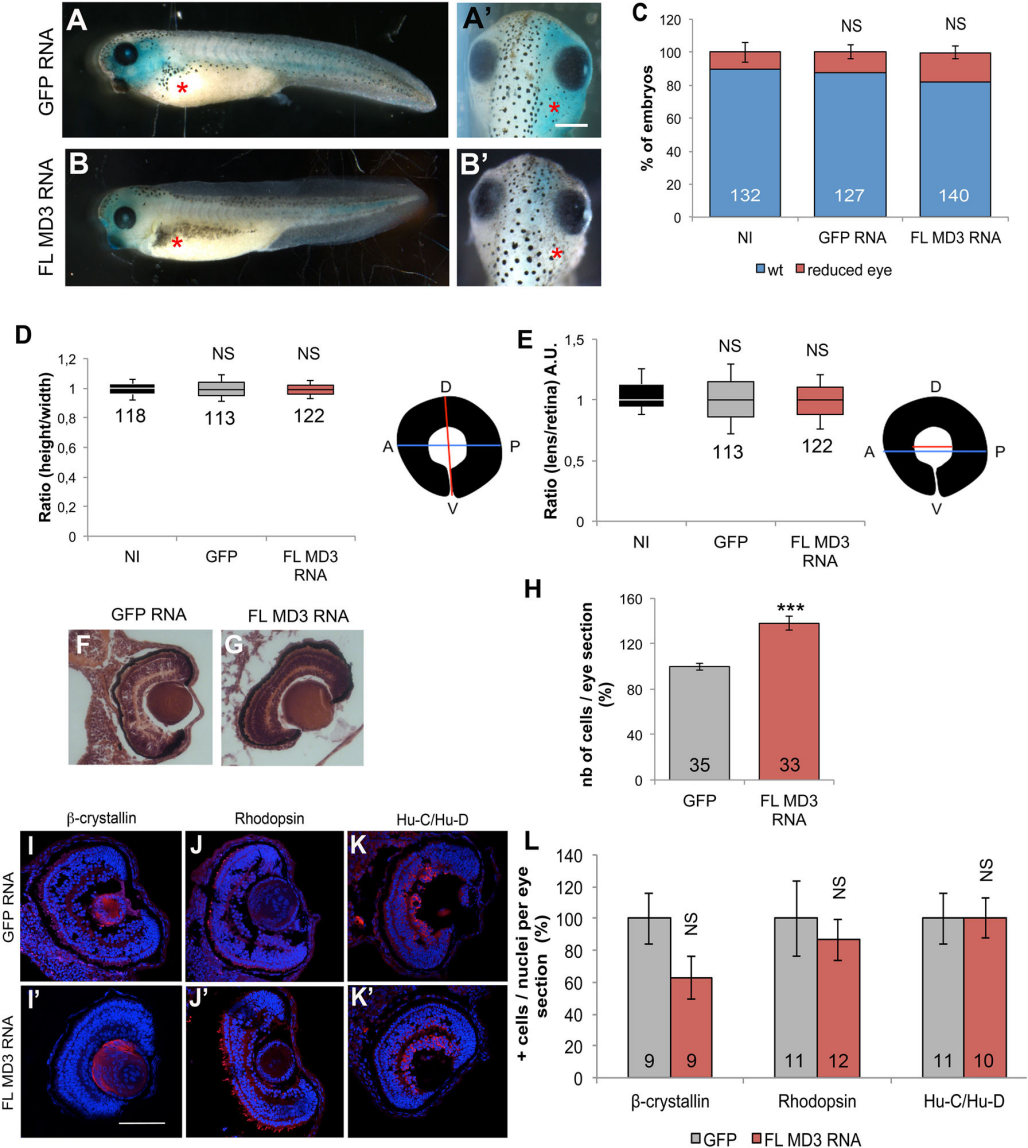
**MD3 overexpression changes eye lamination.** Eye morphology was analysed (A-B′) and quantified (C) in non-injected (NI), GFP or FL MD3 mRNA-injected embryos (*P* values in C, GFP RNA=0.89; FL MD3 RNA=0.26; NS: non significant). Lateral (A,B) and dorsal view (A′,B′) of the embryo; GFP mRNA was used as a control. Red asterisk, injected side of the embryo. (D,E), The eye size was quantified for GFP or FL MD3 mRNA-injected embryos, as described in the schemes and in the Materials and methods. Numbers indicate the embryos analysed; NS, non significant. *P* values in D, GFP RNA=0.66; FL MD3 RNA=0.91; *P* values in E, GFP RNA=0.46; FL MD3 RNA=0.52; NS, non significant. (F,G) Histological analysis by H&E staining was performed on eye sections from GFP and FL MD3 mRNA-injected embryos and the number of cells was quantified as described in the Materials and methods. (H) Number of cell were quantified per eye section stained by H&E derived from GFP or FL MD3 RNA-injected embryos. Numbers indicate the eye sections quantified; *P* value=1.26*10^−6^, ***P<0.001. Immunohistochemistry on eye sections from GFP and FL MD3 mRNA-injected embryos was perfomed for β-crystallin (I-I′), rhodopsin (J,J′), Hu-C/Hu-D (K-K′). (L) Number of positive cells for each eye marker was quantified as described in the Materials and methods. Numbers indicate the eye sections quantified; *P* values, β-crystallin=0.19; Rhodopsin=0.87; Hu-C/Hu-D=0.74; NS, non significant. Statistical significance was assessed by Mann–Whitney (C,L) and Student's *t*-test (D,E,H). Scale bars: 500 µm (A,B) and 100 μm (I-K). Embryos were analysed at stage 42.

As a complementary approach, we injected FL MD3 mRNA to overexpress MD3. MD3 overexpression did not have any evident morphological effects on gastrula, neurula and early tailbud development (Fig. S3). Additionally, MD3 overexpressing tadpoles did not present any visible eye phenotype (Fig. 3A-C), such as altered retinal pigmentation, lens:retina ratio or eye size (Fig. 3D,E). In sections, however, the retina appeared more compact because of an increased number of cells in the MD3 overexpressing-embryos (Fig. 3F-H). However, this change in cell numbers did not affect lens epithelial cells, ganglion cells and rod photoreceptors (Fig. 3I-L). Altogether, these results suggest that MD3 plays a functionally important role in *Xenopus* eye development and morphogenesis.

### MD3 regulates the optic fate of cells-derived from blastomeres A1 and A3

**Fig. 4.**
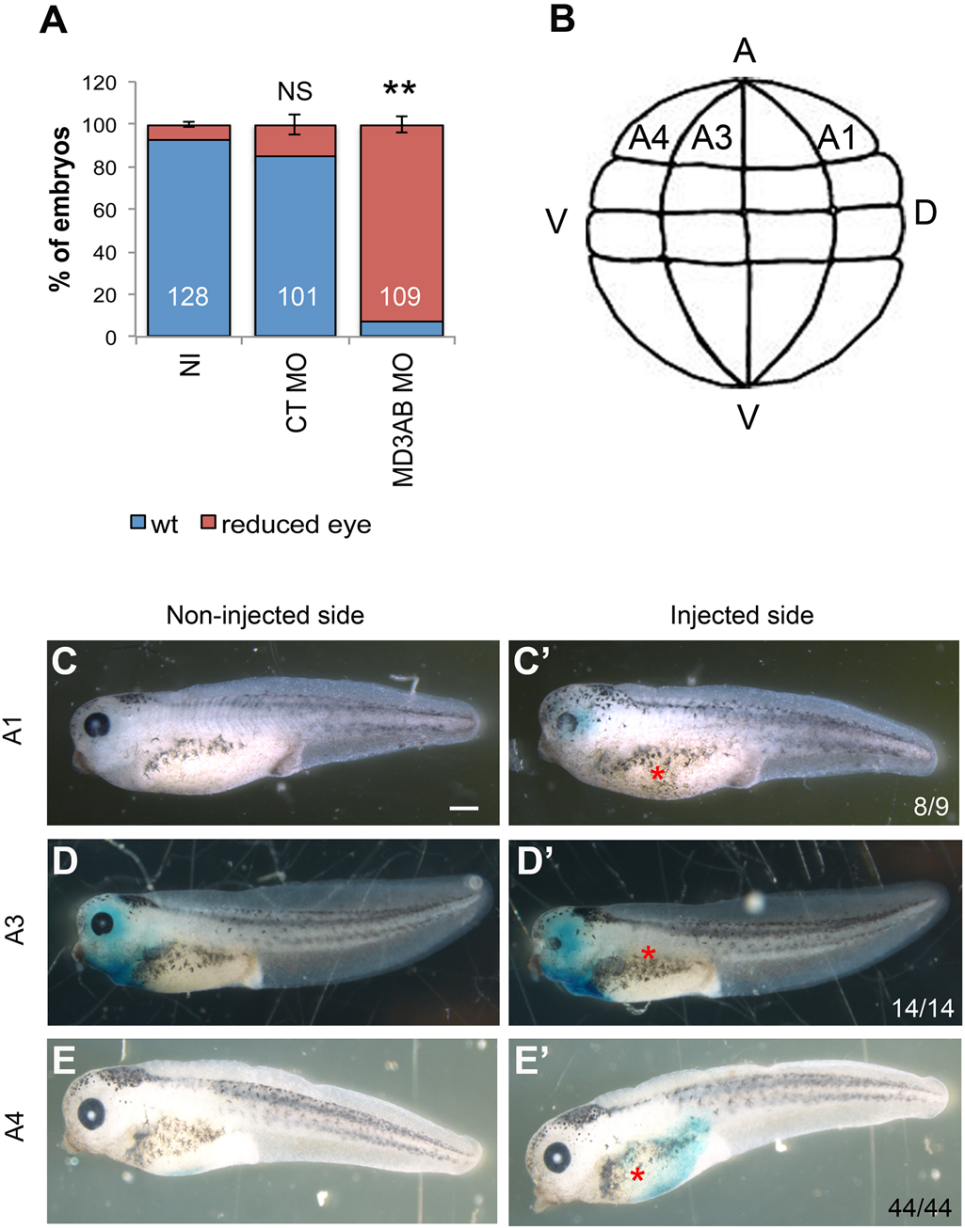
**The MD3 MO-induced eye phenotype originates from neural plate and crest but not mesoderm.** (A) Alteration of eye morphology was quantified in embryos injected with MD3 MOs in one dorsal blastomere at 8-cell stage. NS, non significant; **P<0.01. Student's *t*-test (*P* values, CT MO=0.34; MD3AB MO=1*10^−2^). (B) Scheme representing the dorsal blastomeres A1, A3 and A4 of an embryo at 32-cell stage. A, animal pole; V, vegetal pole; V, ventral; D, dorsal (scheme adapted from Xenbase). Alteration of eye morphology was analysed and quantified in embryos injected with MD3 MOs in one dorsal blastomere A1, A2 or A4 at 32-cell stage (C-E′). Note the disrupted eye morphology in A1- and A3-injected embryos. Scale bar: 500 µm; numbers indicate the embryos analysed at stage 42; red asterisk, injected side of the embryo.

The active Spemann organizer favours normal eye morphogenesis (Bailey et al., 2004; Kim et al., 2005) and convergent extension (Itoh and Sokol, 1999); consequently, the MD3 MO-induced eye phenotype might be due to a dysfunctional mesodermal signal. A mesoderm contribution is unlikely, however, as no shortening of the antero-posterior axis was evident in MD3 morphants (Fig. 2). To rule out the mesoderm involvement, MD3 MOs were injected in a dorsal animal blastomere at the 8-cell stage. This injection also led to the reduced eye phenotype (93%±0.62; Fig. 4A). Hence, the eye phenotype of MD3 depleted embryos is mesoderm-independent.

At 32-cell stage, the eye only derives from nine blastomeres in *Xenopus*, among which the main contributors in the animal pole are (from dorsal to ventral): A1 and A3 for retinal cells, and A1, A3 and A4 for lens cells (Moody, 1987). In addition to their ocular contribution, A1, A3 and A4 blastomeres give rise mainly to the neural ectoderm, neural crest and epidermis, respectively (Fig. 4B). Thus, we asked which of these blastomeres participates in the MD3 MO-eye phenotype by injecting them with MD3 MOs. Fig. 4C-E shows that A1- and A3-injected embryos presented defective eye morphogenesis, but not A4-injected embryos. This suggests that A1 and A3 blastomeres, which mainly develop into neural and neural crest-derived cell types, are responsible for the observed eye phenotype. Hence, MD3 plays an important role during the development of the neural linage that is essential for eye development.

### MD3 controls cell proliferation and survival in the eye field

**Fig. 5.**
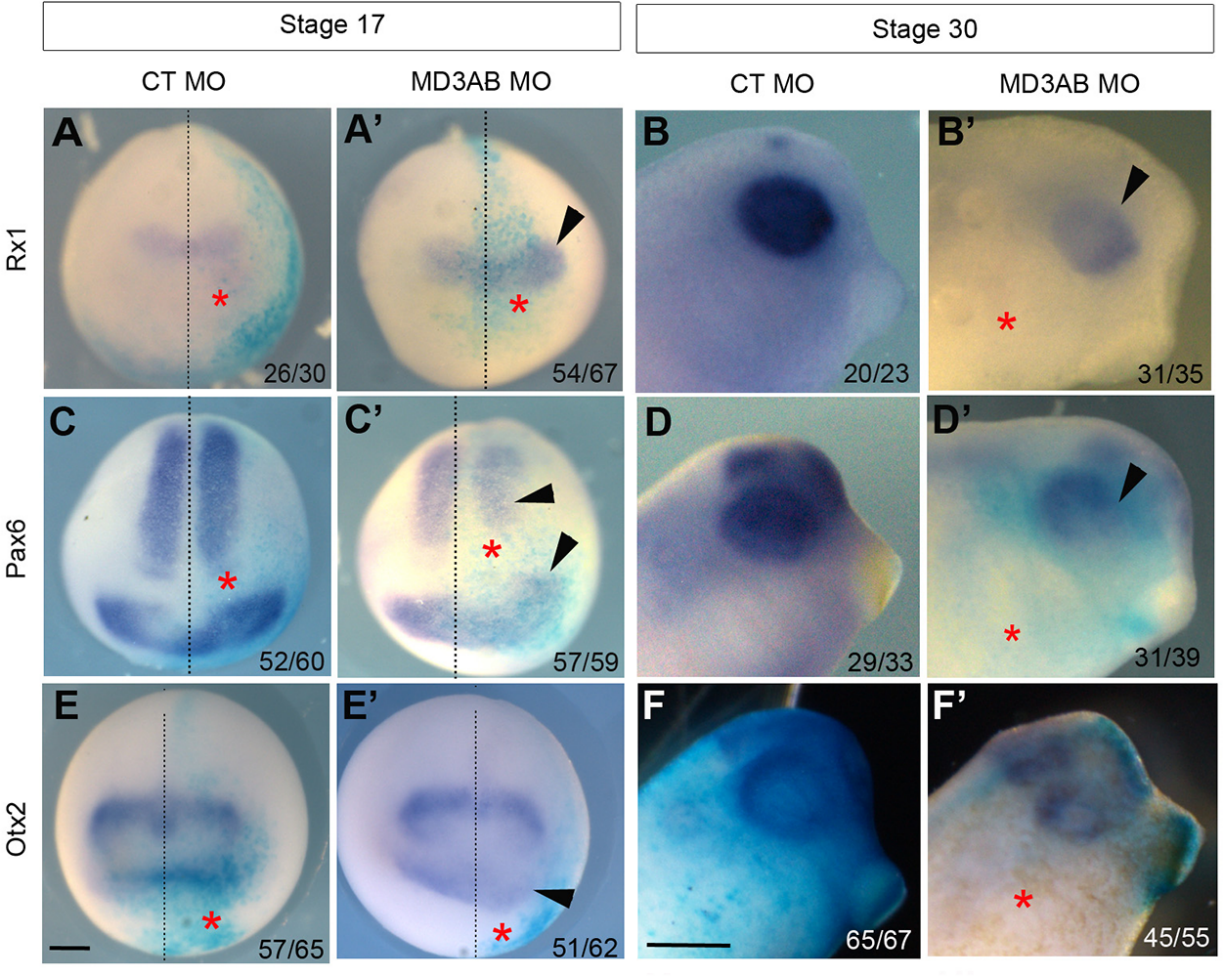
**MD3 KD affects early eye-field formation.** Eye-field specification was analysed by WISH against the eye markers Rx1, Pax6, Otx2 in neurulas (stage 17; anterior view; A,A′,C,C′,E,E′) and tailbuds (stage 30; B,B′,D,D′,F,F′) injected with CT or MD3 MOs. Black dotted line, midline of embryo; arrowhead, enlarged (A′,C′,E′) and reduced (B′,D′,F′) expression domain of the eye markers; red asterisks, injected side of the embryo; numbers indicate the embryos analysed. Scale bar: 500 µm.

**Fig. 6.**
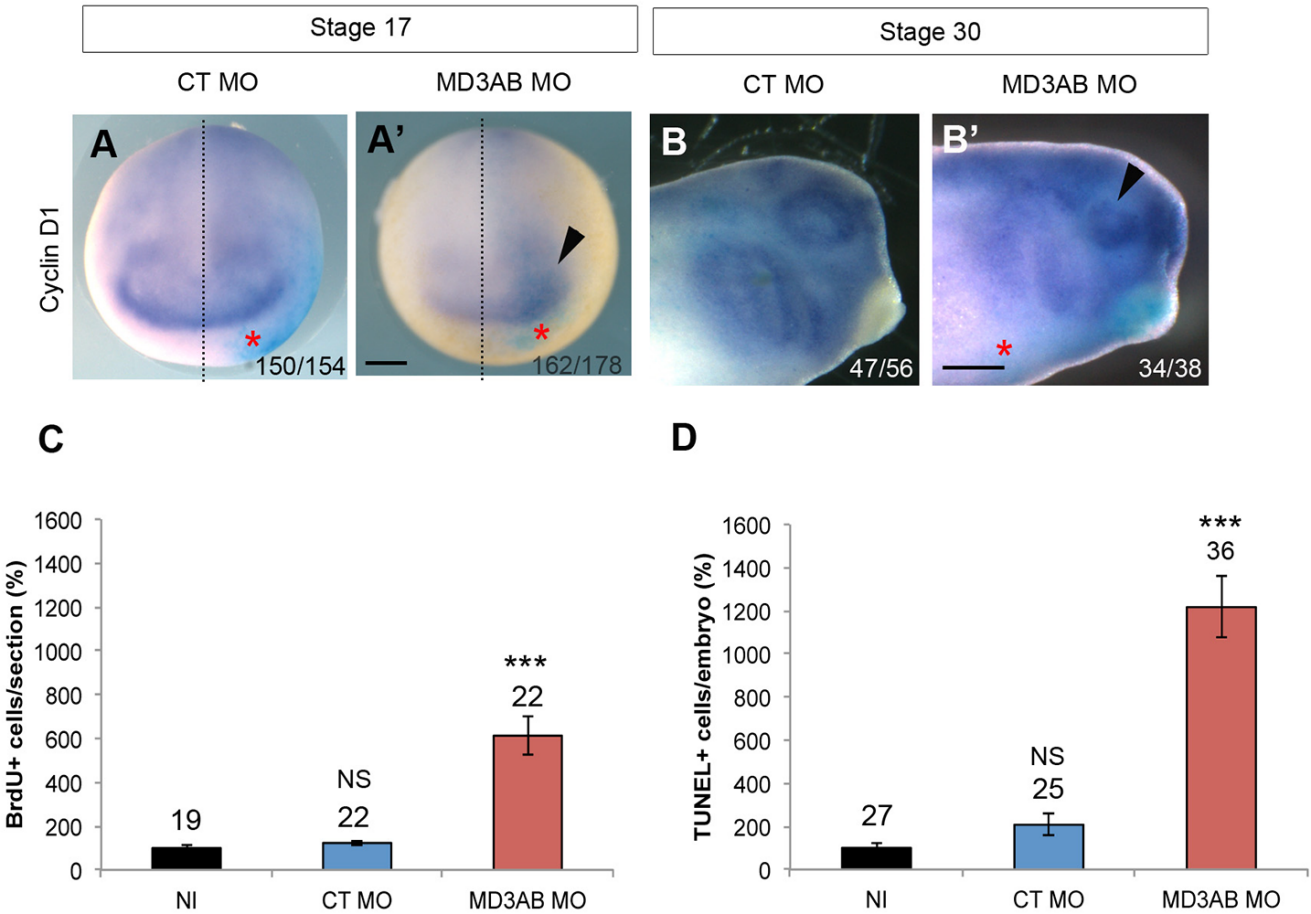
**MD3 KD stimulates cell proliferation and apoptosis in the eye field.** Eye-field proliferation was analysed by WISH against cyclinD1 in neurula (stage 17; anterior view; A-A′) and tailbud (stage 30; B-B′) injected with CT or MD3 MOs. Black dotted line, midline of embryo; arrowhead, enlarged (A′) and reduced (B,B′) expression domain of cyclinD1; red asterisks, injected side of the embryo; numbers indicate the embryos analysed. Scale bar: 500 µm. Cell proliferation in the anterior region of the neurula was quantified with a BrdU staining (C) and cell death with a TUNEL assay in tailbud (D) as described in the Materials and methods; Student's *t*-test (*P* values, BrdU in C, CT MO=0.16, MD3AB MO=9.4*10^−6^; TUNEL in D, CT MO=7*10^−2^, MD3AB MO=5.66*10^−9^). The numbers indicate the embryos analysed (A-B′,D) or the sections counted (C). NS, non significant; ***P<0.001.

Given the contribution of the anterior neural plate region in the eye phenotype, we asked whether MD3 might be necessary for early eye-field formation as a step towards identifying its temporal involvement in eye development. We first analysed by WISH the expression patterns of the EFTFs: retinal homeobox 1 (Rx1), Paired box 6 (Pax6), and Orthodenticle homeobox 2 (Otx2) in neurulas (stage 17) and tadpoles (stage 30) depleted for MD3. Rx1 is a specific eye-field marker, whereas Pax6 is also expressed in the spinal cord and Otx2 at the forebrain/midbrain boundary (Maurus et al., 2005). Rx1 (Fig. 5A-B), Pax6 (Fig. 5C-D) and Otx2 (Fig. 5E-F) expression domains were initially enlarged in the eye-field region of neurulas and then subsequently reduced in tadpoles. These data suggest that MD3 depletion might deregulate the balance between proliferation and survival during early eye-field morphogenesis. To validate this hypothesis, we first established the expression pattern of the cell-cycle marker cyclin D1 and then measured cell proliferation and apoptosis. The cyclin D1 expression domain was enlarged in 91% of embryos at stage 17 and reduced in the eye field of 89.5% of embryos at stage 30 (Fig. 6A-B), indicating that down-regulation of MD3 affected proliferation. Consistently in MD3 morphants, an increase in the number of dividing cells was observed in neurulas (612%±88.52 BrdU-positive cells; Fig. 6C), followed by a massive apoptotic wave in tadpoles (1216.7%±142,6 TUNEL-positive cells; Fig. 6D).

**Fig. 7.**
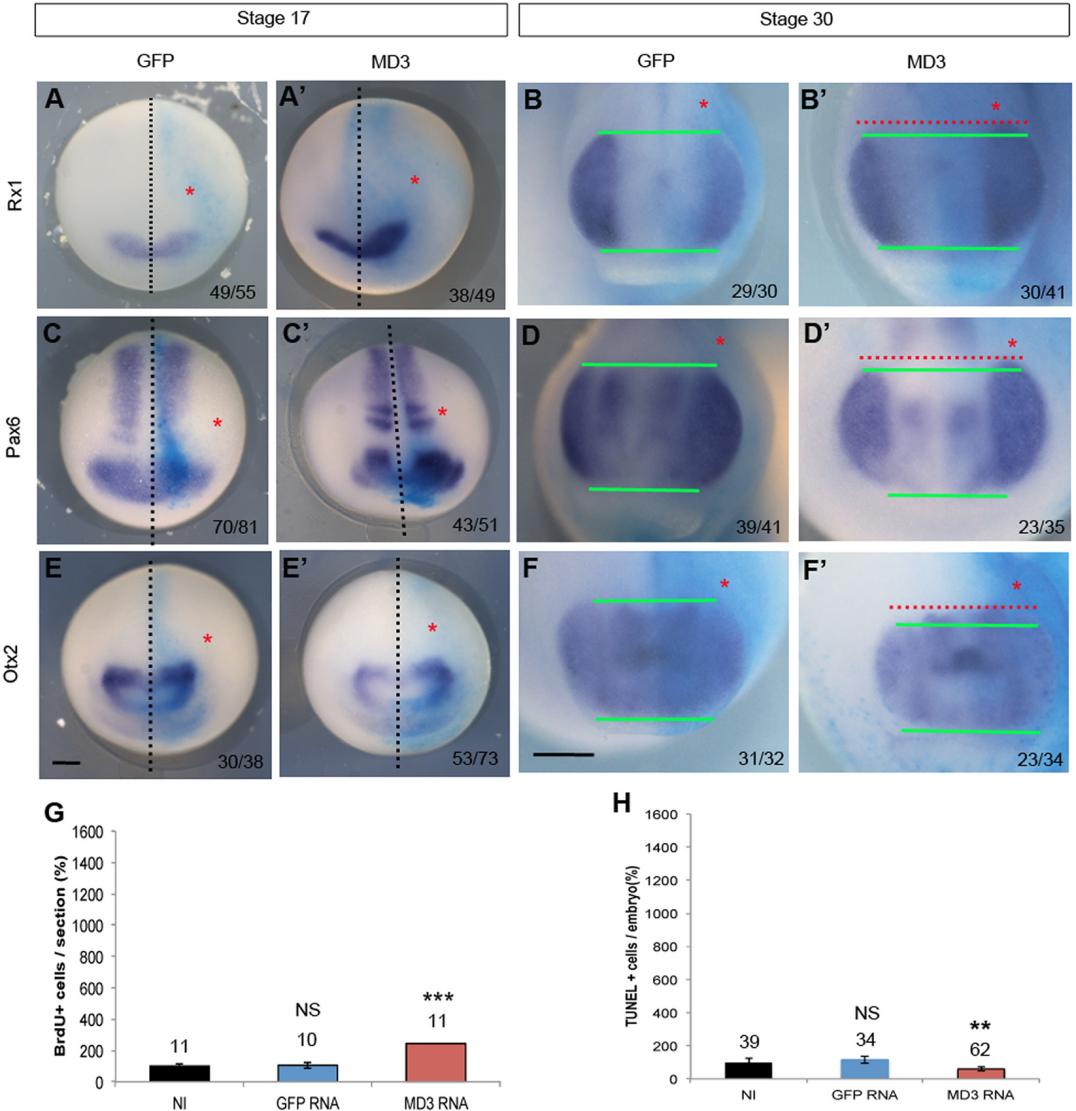
**MD3 overexpression affects the balance between cell proliferation and apoptosis in the eye field.** Eye-field specification was analysed by WISH against the eye markers Rx1, Pax6, Otx2 in neurulas (stage 17; anterior view; A,A′,C,C′,E,E′) and tailbuds (stage 30; B,B′,D,D′,F,F′) injected with GFP or MD3 RNAs. Black dotted line, midline of embryo; green and red lines, enlarged expression domain of the eye markers; red asterisks, injected side of the embryo; numbers indicate the embryos analysed. Scale bar: 500 µm. Cell proliferation in the anterior region of the neurula was quantified with a BrdU staining (G) and cell death with a TUNEL assay in tailbud (H) as described in the Materials and methods; Student's *t*-test (*P* values, BrdU in G, GFP RNA=0.94, FL MD3 RNA=2.1*10^−5^; TUNEL in H, GFP RNA=0.8, FL MD3 RNA=2*10^−3^). The numbers indicate the embryos analysed (H) or the sections counted (G). NS, non significant; **P<0.01 and ***P<0.001.

To reinforce the role of MD3 during early eye morphogenesis, we analysed the impact of MD3 gain-of-function on the expression domains of the EFTFs in neurulas (stage 17), tailbuds (stage 25) and tadpoles (stage 30). Rx1, Pax6 and Otx2 expression domains were mildly enlarged at neurula stage (Fig. 7A,C and E). In tailbuds and tadpoles, we observed a moderate expansion of the eye field (Fig.7B,D,F and Fig. S4). Similarly to our observations in MD3 depleted embryos, these data suggest a potential deregulation of cell proliferation and death. Indeed, the number of proliferating cells was increased in MD3-overexpressing embryos although more modestly than in the MD3 morphants (244% ±19.07 BrdU-positive cells; Fig. 7G). In contrast, apoptosis in tadpoles was reduced (59.3%±10TUNEL-positive cells; Fig. 7H), which is in agreement with the increased cell numbers observed in eye sections. Altogether these data demonstrate that MD3 modulates cell proliferation in the early eye field and regulates cell survival during eye morphogenesis.

### MD3 activates the JNK pathway for eye morphogenesis

Building an eye requires complex regulation of multiple signalling pathways (Zuber, 2010).

Our previous *in vitro* study demonstrated that MD3 acts as a modulator of the JNK pathway and controls gene expression, cell proliferation and survival (Steed et al., 2014). The expression pattern of MD3 *in vivo* is similar to that of JNK and thus raises the question whether MD3 and JNK act together to regulate eye morphogenesis (Garriock et al., 2005). Increased JNK/AP1 activity in MD3 morphants is compatible with the enhanced proliferation observed in the early eye field, however, normal eye morphogenesis is thought to require JNK signalling in *Xenopus* due to activation of a non-canonical Wnt pathway (Lee et al., 2006; Maurus et al., 2005; Valesio et al., 2013). Hence, it could be that MD3 plays a more complex role in JNK pathway regulation than that of a simple repressor.

**Fig. 8.**
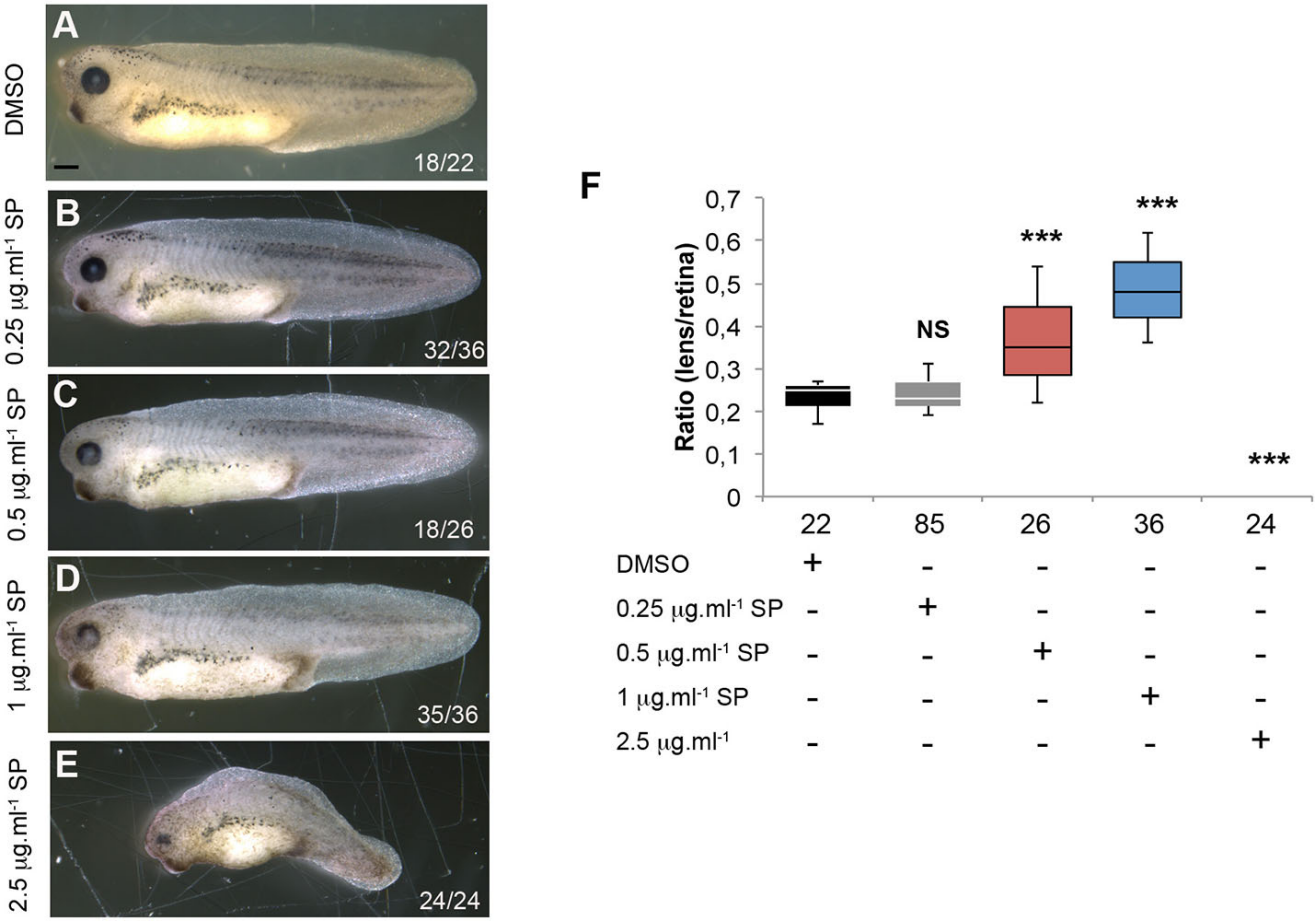
**JNK inhibition disrupts eye morphogenesis.** (A-E) The morphology of the eye was analysed in wild-type embryos treated from stage 11 to 22 with DMSO or increasing concentrations of SP600125, a JNK inhibitor. Scale bar: 500 µm. (F) The eye size was quantified from the embryos (A-E) as described in the Materials and methods; Mann–Whitney test (*P* values, 0.25 μg ml-1=0.93; 0.5 μg ml-1=7.8*10^−5^; 1 μg ml-1=1.02*10^−8^; 2.5 μg ml-1=1.07*10^−5^). The numbers indicate the embryos analysed. Note, the embryos show reduced eye formation in a dose-dependent manner. NS, non significant; ***P<0.001.

To determine the role of JNK downstream of MD3 in eye morphogenesis, we first tested whether inhibition of JNK affects ocular development. We added increasing concentrations of the JNK inhibitor SP600125 before eye field specification at stage 11 and then removed it at stage 22, when the eye field is defined. Modulation of JNK signalling with low concentrations of SP600125 indeed affected eye morphogenesis: the lens:retina ratio increased in a dose-dependent manner (0.25 to 1 μg ml^−1^; Fig. 8). Higher concentrations of the inhibitor disrupted convergent extension, a process in which JNK plays a key role. However, ocular development seems exquisitely sensitive to JNK inhibition as it was already affected at low concentrations of SP600125 that did not seem to affect convergent extension.

Our previous studies indicate that MD3 attenuates the JNK pathway *in vitro* (Steed et al., 2014). Thus, we first treated MD3 MO-injected embryos at concentrations of SP600125 that did not affect convergent extension from stage 11 to 22. JNK inhibition did not rescue the eye phenotype in MD3 morphants, in contrast, the phenotype deteriorated even further (Fig. S5). Hence, a simple model in which MD3 attenuates the JNK pathway during eye development does not appear to be correct.

**Fig. 9.**
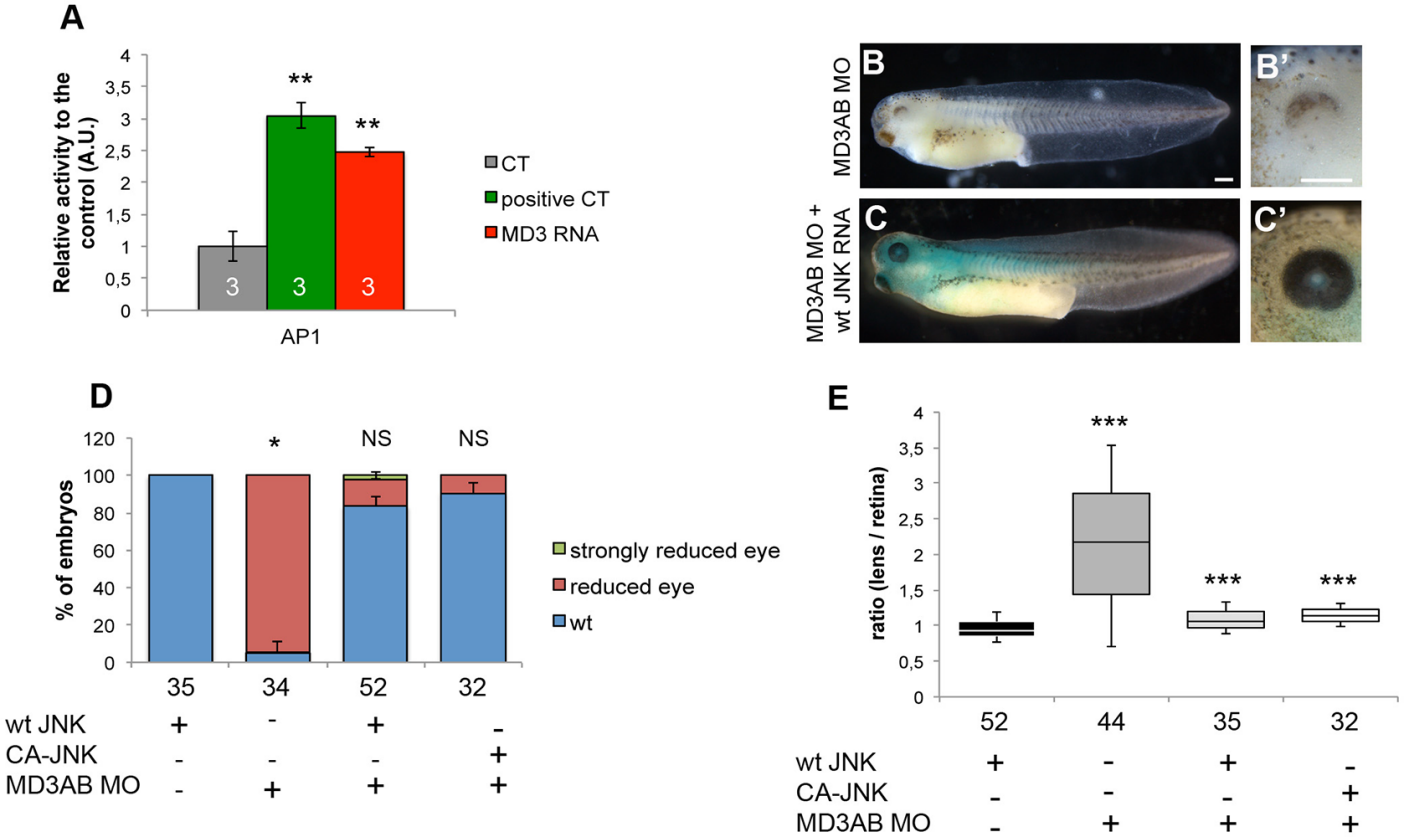
**MD3-induced JNK activation is required for eye morphogenesis.** (A) Activity of the reporter plasmid AP1-Luc (JNK pathway) was determined by Luciferase assay from whole-embryos (stage 12) co-injected with Renilla (as a control), c-Jun (as positive control) or FL MD3 mRNA. The morphology of the eye was analysed at stage 42 (B-C′), quantified (D) and the eye size determined (E) in embryos injected with MD3 MOs, co-injected with wt JNK or CA-JNK mRNA. Scale bars: 500 µm. The numbers indicate the embryos analysed. Statistical significance was assessed by Student's *t*-test (*P* values in A, positive CT=2.6*10^−3^; MD3 RNA=3.5*10^−3^) and Mann–Whitney test (*P* values in D, MD3AB MO=0.02; MD3AB MO+wt JNK RNA=0.06; MD3AB MO+CA-JNK RNA=0.19; *P* values in E, MD3AB MO=1.65*10^−9^; MD3AB MO+wt JNK=1.4*10^−3^; MD3AB MO+CA-JNK=4.37*10^−6^). *P<0.05; **P<0.01; ***P<0.001; NS, non significant.

We next hypothesised that MD3 morphants might have a reduced activity of the JNK pathway perhaps due to JNK deregulation during earlier steps of development. We first asked whether overexpression of MD3 can enhance activity of AP1, a transcription factor stimulated by JNK, in a reporter gene assay in *Xenopus* embryos at stage 12. Fig. 9A shows that overexpression of both c-Jun, used as a positive control, and MD3 increased AP1 activity. Hence, increased MD3 expression can stimulate AP1 activity in some cellular contexts.

If the eye phenotype is caused by reduced activity of the JNK pathway, it should be possible to rescue normal eye development by stimulating JNK signalling. Therefore, we next asked whether co-injecting wild-type JNK (wt JNK) or constitutively active JNK (CA-JNK) could rescue the eye phenotype in MD3 morphants. Indeed, wt JNK and CA-JNK mRNAs rescued the eye phenotype in MD3 morphant embryos (Fig. 9B-E). These data thus suggest that reduced JNK activity in MD3 morphants underlies the eye morphogenesis phenotype.

## DISCUSSION

During *Xenopus* embryogenesis, MD3 is enriched in the lens and plexiform layers, key components for eye morphogenesis. MD3 depletion affected the formation of both the lens and the retina by reducing the number of lens epithelial cells, ganglion cells and photoreceptors, respectively. In the early eye-field, MD3 modulates the balance between cell proliferation and survival, and eye morphogenesis requires activation of the JNK pathway downstream of MD3.

### MD3 is required for eye morphogenesis through JNK pathway activation

MD3 expression strongly recalls the spatial distribution of JNK in *Xenopus* (Yamanaka et al., 2002). The activators of JNK, mitogen-activated protein kinase kinase 7, Plenty of SH3s (POSH), MAP kinase upstream kinase and metastasis-associated kinase (MAK) also present a similar expression pattern (Fuhrmann, 2008; Hirai et al., 2005; Kibardin et al., 2006; Kim et al., 2005; Yamanaka et al., 2002). This similar spatial distribution and the *in vitro* function of MD3 as a JNK regulator support the idea that MD3 also regulates the JNK pathway *in vivo* during the eye morphogenesis.

JNK signalling is activated during embryonic development following exposure to growth or differentiation factors (Valesio et al., 2013). Consequently, several studies linked JNK to eye cell differentiation and eye morphogenesis induced by such factors. Wnt4 depleted embryos lose the expression of EFTFs and develop smaller eyes through the inactivation of the non-canonical Wnt/JNK pathway (Maurus et al., 2005). Interestingly, Kibardin et al. (2006), proved that MAK stimulates the non-canonical Wnt pathway to promote eye morphogenesis in *Xenopus* (Kibardin et al., 2006). Kim et al., (2005) also demonstrated that POSH-induced JNK activation regulates the anterior neural development including eye formation through the control of cell survival (Kim et al., 2005). Our data indicate that MD3 activation of the JNK pathway is required for normal eye development, consistent with these previous studies (Fig. 9). Thus, future detailed analysis of head structure and brain patterning will enable generalizing the mechanism driving eye formation and anterior neural development.

Several studies used the chemical SP600125 to inhibit JNK signalling by preventing c-Jun phosphorylation (Maurus et al., 2005; Valesio et al., 2013; Yamanaka et al., 2002). Zebrafish and *Xenopus* embryos treated with SP600125 (Figs 8 and 9 and Fig. S5) present an underdeveloped retina and lens, strengthening the requirement for JNK during eye morphogenesis. The use of this drug in zebrafish can lead to side effects unrelated to JNK inhibition due to its function as a partial agonist of 2, 3, 7, 8-TetraChloroDibenzo-p-Dioxin (Valesio et al., 2013). However, analysis performed in amphibians showed insignificant side effects (Collier et al., 2008), making it a reliable approach to study the role of JNK.

In addition to the eye phenotype, JNK-inactivated zebrafish embryos also present a reduced anterior-posterior axis corresponding to defective convergent extension movement (Valesio et al., 2013). In *Xenopus*, convergent extension movements and eye development occur via the activation of the non-canonical Wnt pathway and, more precisely, Wnt5 is responsible for JNK activation during convergent extension (Yamanaka et al., 2002). During eye development, the activation of the non-canonical Wnt pathway down-regulates the canonical Wnt signal (Kibardin et al., 2006); this switch is also responsible for eye morphogenesis in zebrafish (Cavodeassi et al., 2005). Altogether these studies corroborate a common origin for eye morphogenesis and convergent extension that might be regulated by the signals from the Spemann organizer. However, the MD3 MO-induced eye phenotype was not due to an effect on the mesoderm (Fig. 4A). Moreover, the length of the embryo was not affected and no dorsal flexure was visible following MD3 depletion (Fig. 2A-C). However, our findings do not exclude an involvement of non-canonical Wnt signalling, and it will be interesting to explore whether MD3 affects the role of JNK in the non-canonical Wnt pathway.

### MD3, a modulator of cell proliferation and survival

JNK regulates cell survival *in vitro* and *in vivo* as a consequence of its increased or decreased activity (Hao et al., 2015; Pushpavalli et al., 2015; Steed et al., 2014; Valesio et al., 2013; Weston and Davis, 2002). This dual-action of JNK suggests a high degree of complexity in the regulation of cell survival depending on the conditions and model analysed. Hence, it is essential to ensure proper temporal control of JNK activity.

At stage 17, increased expression of EFTFs (Rx1 Pax6, Otx2; Fig. 5A-E) and hyper-proliferation characterized MD3 morphants (Fig. 6A,C); followed by increased apoptotic activity (Fig. 6B,D) and a reduced eye field (Fig. 5B-F) at stage 30. Interestingly, overexpression of MD3 in neurula, tailbud and tadpole also led to a mild enlargement of the expression domains of EFTFs (Fig. 7A-F and Fig. S4), correlating with a small increase in proliferating cells (Fig. 7G) and reduced apoptosis (Fig. 7H). Modulation of the eye-field size by manipulation of MD3 expression is thus mirroring a defective balance between proliferation and survival. Disruption of this balance might originate from MD3 control of cell proliferation and survival through JNK activity (Steed et al., 2014) or deregulated expression of cell-cycle activators due to altered levels of EFTFs (Bilitou and Ohnuma, 2010; Zuber, 2010). Cell cycle regulators like cyclin D1 are JNK/AP-1 target genes; hence MD3 may regulate expression of at least some cell cycle regulators via the JNK pathway also in *Xenopus*. *In vitro*, MD3 depletion induces JNK activity, leading to increased proliferation as highlighted by enhanced cyclin D1 expression and AP1 activity (Steed et al., 2014). In this study, we demonstrate that MD3 depletion *in vivo* reduces JNK signalling leading to abnormal eye morphogenesis, and that this phenotype can be rescued by JNK overexpression. However, it is possible that the JNK rescue only affects steps of eye morphogenesis following eye-field specification, as an enlarged eye field, such as the one observed at stage 17, does generally not lead to an eyeless phenotype but rather to giant eyes. The effect of MD3 on the JNK pathway seems to be stimulus and cell-type dependent, and its depletion can thus affect activity of the JNK pathway either negatively or positively. This is supported by the observation that increased MD3 expression in stage 12 embryos resulted in increased in AP-1 activity (Fig. 8A), whereas MD3 expression in cultured cells inhibits AP-1 (Steed et al., 2014).

Another possibility is that MD3 depletion-induced hyper-proliferation is a JNK-independent mechanism and that MD3 depletion disrupts expression of EFTFs, which, in turn, control expression of cell-cycle regulators. Overexpression of EFTFs such as Rx does not only stimulate eye field proliferation but also leads to a giant eye phenotype (Zuber, 2010; Zuber et al., 1999), this suggests that an additional effect induced by MD3 depletion must take place as we obtained smaller eyes or even an eyeless phenotype in MD3 morphants. This additional effect is likely to lead to reduced JNK pathway activity and to take place after the initial induction of the eye field as the smaller eye phenotype was rescued by JNK overexpression.

To conclude, we demonstrate that MD3-induced JNK pathway activation is required for proper eye morphogenesis in *Xenopus*. This study identifies MD3 as an inhibitor of the expression of EFTFs, acting downstream of the inducing signalling molecules; hence, MD3 is a regulatory cornerstone of eye morphogenesis. Further insight into the role of the MD3/JNK pathway during eye development will be required to determine the up- and down-stream signalling mechanisms governing MD3 expression and to unveil the spatio-temporal distribution of active JNK in the eye field.

## MATERIAL AND METHODS

### *Xenopus laevis* and drugs

Animals were purchased from Nasco (Salida, California), used under Act 1986 (UK Home Office) according to the UK Home Office ethical approval and guidance and University Animal Welfare Committee. The embryos were staged using Nieuwkoop tables (Nieuwkoop and Faber, 1994). DMSO (dimethyl sulfoxide; Sigma) or SP600125 (Tocris) was added at the indicated concentrations during the growth of embryos (stage 11 to 22).

### Semi-quantitative RT-PCR

To determine xMD3 expression, total RNA from 40 optic vesicles [stage 25; without staining as described in McDonough et al. (2012)] and 50 eyes (stage 42; removal of epidermis in rostro-caudal manner and eye isolation) was extracted following the manufacturer instructions (RNeasy mini kit, Qiagen) and a semi-quantitative PCR was performed for MD3 and GAPDH, with the following primers respectively: MD3 Fw, ACAATGAGAGATTCTGTTACAGACGGGG; MD3 Rv, AAGCTCCCAACAGTTACAGCATCCATGGCT; GAPDH Fw, ACTGCCACCCAGAAGAC; GAPDH Rv, AAGTGCTTATTCCTTAGATG.

### DNA constructs

MD3 antisense probe was generated by amplifying the MD3 ORF 3′-end using the following primers, Fw: CCCGGGGGATCCATGAGAGATTCTGTTACAGACG, Rv: CGGTGGCGGCCGTCAGCAGATCAGGATCAGC and inserted in pBluescript BamH1/Eag1 restriction sites. MD3 overexpression and rescue constructs were obtained by subcloning GFP tagged-full-length MD3 (GFP FL MD3) in pCS2+ (BamH1/EcoR1; Fw: GTTCGCGGATCCGCCACCATGGTGAGCAAGGGCGAG; Rv: CTTCCGGAATTCTCAAACATAGTTGTTGGGTTTCTTTTTTAAC) and a mutant MD3 (7mut-MD3) in pCS2+ (BamH1/EcoR1; Fw: GTTCGCGGATCCGCCACCATGAGgGAcTCaGTaACtGAtGGcGAGAATCGGGCACCTAG; Rv: CTTCCGGAATTCTCAAACATAGTTGTTGGGTTTCTTTTTTAAC; mutated bases are in lower cases).

### Whole-mount *in situ* hybridization (WISH) and morpholinos

The embryos were fixed in MEMFA [0.1 M MOPS (3-N-morpholino) propane-sulfonic acid], 2 mM EGTA (ethylene glycol tetra-acetic acid), 1 mM MgSO4, 3.7% formaldehyde. X-gal staining and WISH were performed as described in Luehders et al. (2015). Control (CT), Morpholino (MO) and MarvelD3 (MD3) MOs were purchased from Gene Tools (CT MO: CCTCTTACCTCAgTTACAATTTATA; MD3A MO: AGACCCAAATCTTCCTTTTGTTCCC; MD3B MO: CCCGTCTGTAACAGAATCTCTCATT). MD3A MO was designed to target the 5′-UTR (untranslated region) of MD3, while MD3B MO sequence overlapped the start codon of MD3 ORF (open reading frame).

### Histology, immunocytochemistry and immunoblot

For Hematoxylin and Eosin (H&E) histological staining, embryos (stage 42) were fixed 1 h at room temperature in MEMFA and equilibrated in 30% sucrose, before embedding in OCT compound (Tissue Plus, Scigen, USA) for cryo-sectioning (thickness: 8 μm; Leica). For immunocytochemistry, the embryos were rinsed once in water, fixed overnight in 4% PFA at room temperature for β-crystallin staining or 1 h in MEMFA at room temperature, followed by an overnight incubation at −20°C in Dent's fixative (4 volumes methanol:1 volume DMSO) for Rhodopsin and Hu-C/Hu-D staining. Next, the embryos in Dent's fixative were rehydrated, equilibrated in 30% sucrose at room temperature, and embedded in OCT compound before cryosectioning (thickness: 8 μm, Leica). The eye sections were post-fixed in 4% paraformaldehyde, blocked in PBST [0.3% Triton X100 in PBS (phosphate buffer saline)] with 10% goat serum or bovine serum albumin (BSA), then incubated with the following primary antibodies: monoclonal mouse anti-β-crystallin (Abcam; ab90379), mouse anti-Hu-C/Hu-D (Life technologies; A21271) and polyclonal rabbit anti-Rhodopsin (H-100; sc-15382, Santa Cruz) antibodies. For immunoblots, 20 ACs were homogenized in RIPA lysis buffer (50 mM Tris-HCl pH8, 150 mM NaCl, 1% Nonidet 40, 0.5% sodium deoxycholate, 0.1% SDS, cocktail of protease inhibitors). Protein expression was then determined with polyclonal anti-GFP (Invitrogen) and anti-Actin (A2066, Sigma Aldrich) antibodies.

### Proliferation and apoptosis assays

To assess cell proliferation, the embryos were injected with BrdU (30 nl of 10 mM 5-Bromo-2′-deoxyuridine) in the anterior neural tube, harvested 2 h later (stage 17) in MEMFA and processed for cryo-sectioning. Sections were stained as described in Auger et al. (2012). To determine cell death, embryos (stage 30) were fixed in methanol and processed for a whole-mount TUNEL (terminal deoxynucleotidyl transferase dUTP nick-end labelling) assay (http://tropicalis.berkeley.edu/home/gene_expression/TUNEL/TUNEL.html).

### Reporter assay

The promoter activity of AP1-Luc plasmid was determined by co-injection with pRL-CMV (Promega; as a normalization control), c-Jun RNA (positive control) and FL MD3 RNA. Embryos (stage 12) were processed as triplicates for the reporter assay following the instructions of the manufacturer (Dual Luciferase reporter assay; Promega).

### Imaging

A SMZ 1500 Nikon microscope (Apochromat HR Plan 0.5×/W. D. 136 mm objective; camera Digital Sight DS-Fi2; Nikon) was used for morphological analyses, while a LSM710 confocal microscope (C-Apochromat 40×/1.2 N.A. objective; Zeiss) was used for immunocytochemistry on eye sections. All images were analysed with ImageJ (https://imagej.nih.gov/ij/).

### Quantification and statistics

Specific cell types in the eye were quantified by determining the ratio of the number of cells positive for a given eye marker divided by the number of nuclei in the same eye section. The total number of cells corresponds to the number of nuclei per eye section visualized by Hoechst staining. The eye size corresponds to the ratio of the injected side_(height/width)_:non-injected side_(height/width)_ in Fig. 3D, and ratio of the injected side_(lens/retina)_:non-injected side_(lens/retina)_ in Figs 3E, 8F, 9E, and Fig. S5D. The height and width correspond to the dorso-ventral and the antero-posterior length of the retina, respectively. The lens and retina were measured along the antero-posterior axis of the eye. The eye-field transcription factors expression domain area corresponds to the ratio of the injected side:non-injected side in Fig. S4. All measurements were performed with ImageJ. We quantified the number of BrdU-positive cells per section of the anterior region of the embryo for the proliferation assay (Figs 6C and 7G) and the number of TUNEL-positive cells per embryo for the apoptotic assay (Figs 6D and 7H). Error bars indicate s.e.m.; *P* values (NS, non-significant; *P<0.05; **P<0.01; ***P<0.001) were determined based on three independent experiments using Mann–Whitney or Student's *t*-test.
